# Recent advances in understanding and managing contact dermatitis

**DOI:** 10.12688/f1000research.13499.1

**Published:** 2018-06-20

**Authors:** Stefan F. Martin, Thomas Rustemeyer, Jacob P. Thyssen

**Affiliations:** 1 Allergy Research Group, Department of Dermatology, Faculty of Medicine, University of Freiburg, Freiburg, D-79104, Germany; 2 Department of Dermatology, VU University Medical Centre (VUmc), De Boelelaan 1117, Amsterdam, 1081HV, Netherlands; 3 Department of Dermatology and Allergy, Herlev and Gentofte Hospital, Hellerup, DK-2900, Denmark

**Keywords:** contact dermatitis, chemical, skin, treatment

## Abstract

About 20% of the general population is contact-sensitized to common haptens such as fragrances, preservatives, and metals. Many also develop allergic contact dermatitis (ACD), the clinical manifestation of contact sensitization. ACD represents a common health issue and is also one of the most important occupational diseases. Although this inflammatory skin disease is mediated predominantly by memory T lymphocytes recognizing low-molecular-weight chemicals after skin contact, the innate immune system also plays an important role. Along that line, the presence of irritants may increase the risk of ACD and therefore ACD is often seen in the context of irritant contact dermatitis. In this review article, we discuss recent progress in basic research that has dramatically increased our understanding of the pathomechanisms of ACD and provides a basis for the development of novel diagnostic and therapeutic measures. Current methods for diagnosis as well as treatment options of ACD are also discussed.

## Introduction

Allergic contact dermatitis (ACD) is an inflammatory skin disease that affects about 20% of the adult general population and is also an important occupational skin disease ^1– 3^. A recent study showed that 27% of the general population from five European countries had contact allergy (that is, sensitization to at least one contact allergen of the European baseline series) ^4^. A large proportion of these individuals are at risk of developing ACD following exposure to everyday products. Among the occupational diseases, 40% are skin-related ^5^. Contact dermatitis (both irritant and allergic) accounts for about 90% of these. Collectively, these epidemiological data demonstrate the importance of ACD as a challenge to human health. Therefore, basic and clinical research is needed to understand the pathomechanisms of ACD and to develop better strategies for diagnosis and treatment.

**Figure 1.**
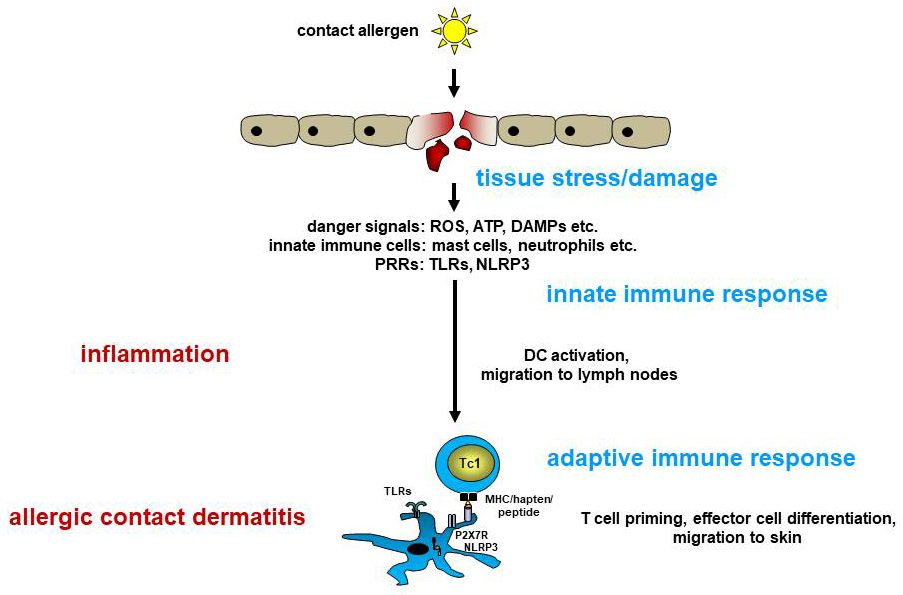
Sensitization phase of allergic contact dermatitis. Contact allergens penetrate the skin and cause tissue stress and damage. Reactive oxygen species (ROS) are formed, ATP is released from stressed cells, and damage-associated molecular patterns (DAMPs) are formed/released from cells. DAMPs then trigger activation of the innate immune system via Toll-like receptors (TLRs) and NOD-like receptor pyrin containing 3 (NLRP3). This results in skin inflammation and consequently activation of dendritic cells (DCs) and migration to the skin-draining lymph nodes. DCs present contact allergens to naïve T cells, leading to their activation and effector cell differentiation. This concludes the sensitization phase. In the elicitation phase, repeated skin contact with the same contact allergen induces inflammation, and T cells are recruited into the inflamed skin, where they exert their effector functions, leading to clinical symptoms of allergic contact dermatitis. MHC, major histocompatibility complex; PRR, pattern recognition receptor.

ACD is mediated by T cells recognizing low-molecular-weight organic chemicals or metal ions in the context of major histocompatibility complex (MHC) molecules ^6^. These usually electrophilic chemicals penetrate the skin and react with extracellular and cellular proteins. Their protein reactivity is mandatory and underlies their unusual ability to trigger innate immune as well as T-cell responses ^7– 9^. Activation of the innate immune system is a prerequisite for the activation and skin migration of contact allergen-specific T cells. The first skin contact with allergens initiates the activation of skin cells, most importantly the epidermal Langerhans cells and dermal dendritic cells (DCs), which subsequently migrate to the local lymph nodes and present the contact allergen(s) to naïve T cells. Contact allergen-specific T cells then proliferate and differentiate to effector T cells that enter the blood circulation. Repeated skin contact with the same contact allergen then results in the recruitment of these T cells into the skin and the elicitation of the clinical reaction of ACD ( Figure 1). The response is limited and downregulated by regulatory T and B cells, natural killer T (NKT) cells, and further cell types ^9^. Here, we review recent progress in basic research aimed at understanding the cellular and molecular mechanisms underlying the innate immune responses as well as the pathogenic T-cell response and its regulation. Moreover, we will give an overview of the current status of the management of ACD in the clinic.

## Protein reactivity of contact allergens

The central role of the protein modification by contact allergens is underlined by the fact that two of the three validated *in vitro* assays for the identification of contact allergens are based on protein reactivity. The Direct Peptide Reactivity Assay—Organisation for Economic Co-operation and Development (OECD) test guideline TG 442C—detects the depletion of model peptides containing modifiable lysine or cysteine residues. Electrophilic chemicals binding covalently to the ε-amino group of lysine or the thiol (SH) group of cysteine are classified as potential contact allergens. Similarly, covalent modification of cysteine residues in the cytosolic protein Keap1 and the subsequent release and DNA binding of the transcription factor Nrf2 lead to the activation of luciferase in HaCaT keratinocytes in the ARE-Nrf2 Luciferase Test Method (OECD test guideline TG 442D) ^10^. The Keap1/Nrf2 pathway is central in the antioxidant phase 2 response.

One of the most challenging research questions is the identification of (a) the target proteins that, upon hapten modification, lead to activation of the innate immune system and cellular stress responses and (b) the natural T-cell epitopes generated after protein modification. Until now, the few known physiologically relevant protein targets of contact allergens have been identified in cell lines. Recent studies addressing these questions *in vivo* have identified keratins in mouse skin after topical application of bromobimanes ^11^ and the macrophage migration inhibition factor (MIF) in skin-draining lymph nodes after topical application of tetramethylrhodamine isothiocyanate (TRITC) ^12^ as protein targets for hapten modification. It is still not known whether these hapten modifications alter the function of the proteins or generate T-cell epitopes. In the case of MIF, an N-terminal proline modification was detected, but potential effects on the function of MIF were not investigated.

## Role of mast cells and neutrophils in contact hypersensitivity

Skin inflammation is essential in the sensitization and elicitation phase of ACD. Its initiation requires the close cooperation of different cell types, which together orchestrate this complex response ( Figure 1). It was recently demonstrated that mast cells are important innate effector cells in murine contact hypersensitivity (CHS), the mouse model for ACD ^13^. CHS was significantly reduced in mast cell-deficient or -depleted mice. The reasons for that were the lack of neutrophil extravasation into inflamed skin initiated by the localization of mast cells in proximity of blood vessels and by secretion of the pre-formed and *de novo* synthesized neutrophil-attracting chemokines CXCL1/CXCL2 as shown in a lipopolysaccharide-induced peritonitis model. Interestingly, macrophages were required for deeper neutrophil migration into the tissue ^14^. Granule release and *de novo* chemokine synthesis were Toll-like receptor 4 (TLR4)-dependent. DC emigration from the skin was compromised by the selective absence of mast cells or neutrophils, as was T-cell recruitment to the skin ^13– 15^.

A very interesting study in a model of chronic CHS showed that mast cells can also limit CHS ^16^. Using mast cell-deficient Sash or mast cell-depleted Mcpt5-Cre diphtheria toxin receptor (DTR) mice, the authors showed a significant increase of the ear swelling reaction in mast cell-deficient mice which was dependent on CD8 ^+^ tissue-resident memory T (T _RM_) cells. The effect correlated with elevated levels of interleukin (IL)-15 needed for cutaneous T _RM_ cells. Thus, in this chronic CHS model, mast cells limited the CD8 ^+^ T _RM_ cell response by degrading IL-15 via proteases such as chymase and carboxypeptidase A.

## Role of tissue-resident T cells and γδ T cells in contact hypersensitivity

In general, T _RM_ cells form local memory in tissues and are responsible for rapid and strong reactions upon re-exposure to a contact allergen. It was shown that T _RM_ cells are generated from the same naïve precursor as central memory T (T _CM_) cells in the skin-draining lymph nodes. They seed the contact allergen-exposed skin sites, reside there, and produce a rapid allergic response upon re-exposure ^17^. Schmidt *et al*. identified IL-17- and interferon-gamma (IFN-γ)-producing CD8 ^+^ T _RM_ cells in the 2,4-dinitrofluorobenzene (DNFB) CHS mouse model ^18^. The generation of local memory due to T _RM_ cells was shown for both mice and nickel-allergic humans.

It was recently reported in the CHS model that dendritic epidermal T cells (DETCs), which are not found in human skin, rapidly produced IL-17 in response to contact allergens ^19^. However, how these cells were activated was unclear. Nielsen *et al*. revealed that the activating natural killer (NK) receptor NKG2D is involved in their activation ^20^. NKG2D was found to be expressed on most DETCs and in human CLA ^+^ γδ T cells. Mouse keratinocytes upregulated various stress-induced NKG2D ligands when exposed to contact allergens *in vitro*. Moreover, in the mouse system, the NKG2D ligand Mult1 was upregulated by contact allergens in the skin. Experiments with anti-NKG2D blocking antibodies showed a partial block in the activation of DETCs. This study suggests a contact allergen-induced interaction of epidermal γδ T cells with keratinocytes which results in an IL-1β-driven and NKG2D/NKG2DL-dependent T-cell activation and production of IFN-γ and IL-17. This may be part of the early antigen non-specific innate inflammatory response to contact allergens. Jiang *et al*. recently reported a role for dermal γδ T cells in promoting CHS by IL-17-dependent neutrophil recruitment ^17^. They identified a population of γδ T cells which had characteristics of tissue-resident cells with low re-circulation potential. These dermal γδ T cells produced IL-17 and IL-22. Importantly, DNFB-induced CHS was significantly reduced in mice selectively lacking dermal γδ T cells but not DETCs. The authors showed that this was due to reduced neutrophil recruitment. This study underlines the important role of neutrophils in CHS ^15^.

Much progress has been made in the field of metal allergies. Since nickel and cobalt were identified as the first contact allergens able to directly activate a pattern recognition receptor (that is, human TLR4 by inducing its dimerization and signaling ^21– 23^), it was shown that nickel also activates the NLRP3 (NOD-like receptor pyrin containing 3) inflammasome ^24^. Likewise, chromium VI, but not chromium III, compounds activate the NLRP3 inflammasome ^25^. However, chromium fails to provide a priming signal, such as TLR4 activation in the case of nickel, which is needed for inflammasome activation. This was shown *in vitro*, and the findings are reminiscent of contact allergens such as 2,4,6-trinitrofluorobenzene (TNCB) and oxazolone which also fail to do that *in vitro* ^26, 27^. In that case, a tissue-derived priming signal was generated by induction of hyaluronic acid breakdown ^27^. Thus, it may well be that chromium also has to induce a tissue-derived priming signal.

Recently, TLR3 was shown to modulate CHS responses in mice. Both irritant contact dermatitis (ICD) (croton oil) as well as ACD (TNCB) were reduced in the absence of TLR3 in knockout (KO) mice and increased in TLR3-overexpressing mice ^28^. For ACD to TNCB, a role for TLR3 was confined to the elicitation phase. The mechanism of TLR3 activation was not identified, but the authors speculated on the release of self-RNA from necrotic skin cells. It must also be considered that TLR3 ligands may derive from the bacterial skin flora. Experiments in germ-free mice would be needed to clarify that. Up to now, CHS experiments in germ-free mice have indicated that the innate immune system activation can be triggered by contact allergens in the absence of a microbial flora ^26, 27^.

In general, the data published up to now for organic chemical allergens and metal allergens in humans and mice reveal a common mechanistic innate immune response pathway. Contact allergens generate tissue-derived activators of TLRs or are themselves direct activators. Following TLR activation, pro-inflammatory mediators, among them pro-IL-1β, a central cytokine in ACD, are produced. The NLRP3 inflammasome is then activated by different means depending on the contact allergen. Oxidative stress that promotes inflammation is induced ( Figure 1). In general, TLR triggering and inflammasome activation are essential steps in the innate immune response and the mechanisms underlying their activation present as variations of a common theme. It remains to be determined how general this theme is given the high number and physicochemical diversity of the more than 4,000 contact allergens known today.

Skin inflammation can also be induced by irritant chemicals such as detergents like sodium dodecyl sulfate (SDS). This is due in part to a damaging effect on the skin barrier. Therefore, a combination of irritants and contact allergens as often found in cosmetics, household products, and workplace materials can facilitate sensitization due to the amplification of skin inflammation resulting in, for example, the augmentation of DC activation ^29^. Moreover, combinations of contact allergens show similar augmentation due to additive or synergistic effects of the so-called irritant effects of contact allergens (that is, their ability to activate the innate immune system) ^9, 30– 32^.

## Genomics and proteomics

The identification of biomarkers would be very helpful in improving diagnostics and treatment of ACD ^33^. Dhingra *et al*. performed gene array studies using skin samples from patch test biopsies ^34^. Besides identifying 149 genes that were differentially expressed in all contact allergen-treated samples, they identified a significant number of genes that were regulated in a contact allergen-specific manner. This study nicely shows that the clinical appearance of ACD can be very similar for different contact allergens but that the underlying immune responses can be very different as highlighted here for the polarization of the T-cell response.

Quaranta *et al*. compared different forms of eczema (atopic and non-atopic) regarding gene expression profiles ^35^. They made use of an interesting study population: patients with both psoriasis and eczema. In addition, some of these patients developed ACD when tested with nickel. This study allowed an inter-individual comparison of different types of eczema which could be differentiated on the basis of their characteristic gene expression. Comparing naturally occurring eczema with nickel-induced eczema revealed that 33 genes were commonly regulated but that 172 genes were exclusively regulated in induced and 28 exclusively in naturally occurring eczema. Pathway analysis revealed marked differences in genes regulating epithelial differentiation, extracellular matrix, cell–cell adhesion, and the acute immune response. Examples for ACD-specific genes were genes for T-cell attraction ( *CXCL9*, *10*, and *11*) that had already been described as discriminators for ACD ^36, 37^, LCE1 and LCE2 family genes, and *HAS3*, *EPSTI1*, *ICAM-1*, *CXCL8*, *IL-1β*, and *AIM2*. Interestingly, detailed data analysis led the authors to claim a two-gene classifier ( *NOS2* and *CCL27*) for the distinction between psoriasis and atopic dermatitis ^38^.

Besides analysis of gene expression, proteomics are being used to identify contact allergen-induced changes in protein profiles. Using matrix-assisted laser desorption/ionization-mass spectrometry analysis, Jakob *et al*. showed that Ni ^2+^ treatment of human CD14 ^+^ monocytes isolated from peripheral blood mononuclear cells significantly altered the expression of 56 protein species ^39^. Further analysis revealed the induction of proteins associated with apoptotic cell death at concentrations of around 250 µM and above, concentrations which did not affect T-cell viability. Interestingly, Schmidt *et al*. had previously shown a sensitization of human umbilical vein endothelial cells for TRAIL (tumor necrosis factor-related apoptosis-inducing ligand)-induced cell death by nickel ^40^.

Mussotter *et al*. compared the protein profiles of bone marrow-derived dendritic cells (BMDCs) from C57BL/6 wild-type and Nrf2-deficient mice ^41^. More than 50 proteins were upregulated upon BMDC treatment with cinnamic aldehyde and more than 30 upon 2,4-dinitrochlorobenzene (DNCB) treatment. Notably, almost all of these proteins were not upregulated in BMDCs from Nrf2-deficient mice. Nrf2-dependent proteins were associated with oxidative stress, cell survival/death, proteostasis, and other signaling pathways. In addition, metabolic reprogramming by contact sensitizers was revealed (for example, by upregulation of many glycolytic enzymes). This approach allows identification of Nrf2-dependent and -independent proteins and differentiation between contact sensitizer- and irritant-specific effects.

More recent profiling studies of patients with ACD used serum ^42^ samples from stratum corneum after tape stripping ^43^, skin biopsies ^44, 45^, or monocytes ^36^. Analyses were carried out by protein or gene arrays and mass spectrometry. Owing to the diversity of the samples, the different contact allergens used, and the different methods used, it is impossible to compare the data and search for common biomarkers. Nevertheless, inflammation and skin barrier-related genes and proteins are, not surprisingly, differentially regulated in all studies. The big challenge is the identification of biomarker profiles that allow differentiation between ACD and ICD and between ACD and other forms of eczema.

More such genomic and proteomic studies are needed to understand how contact sensitizers work on a mechanistic basis. Novel biomarkers can be identified that may aid in the improvement of ACD diagnostics, identification of novel drug targets, and *in vitro* assay development for contact allergen identification and replacement of animal testing.

## Immune regulation and tolerance to contact allergens

Regulatory T cells (Tregs) are central regulators of the extent and duration of CHS responses. The important role of Tregs in CHS has been underlined by a recent study using CD103-deficient KO mice ^46^. The integrin αE (CD103) is expressed on subsets of DCs and on Tregs localizing to epithelia. CHS to oxazolone or DNCB was significantly increased in the KO mice. The authors then found that the level of Foxp3 was significantly reduced in CD4 ^+^CD25 ^+^ T cells and that Tregs from the KO mice were unable to suppress CHS. This study shows that CD103 has a role in the regulation of Foxp3 expression in addition to its function of retention of Tregs in inflamed skin.

Tregs express the ectonucleotidases CD73 and CD39, which degrade ATP to tolerogenic adenosine ^47^. It has been shown that one consequence of this is the retention of CD8 ^+^ effector T cells in the lymph nodes. Mahnke *et al*. showed that CD8 ^+^ T cells shed CD62L in order to leave the lymph node, a process required for effector cell migration to inflamed skin in CHS ^48^. ATP is released from skin cells following contact allergen application ^49^. In T cells, ATP acts on P2X7R to upregulate ADAM17 which sheds CD62L from the T-cell surface. Moreover, CD73-dependent ATP degradation seems to limit the extent of DC emigration from skin and eventually the magnitude of CHS ^50^.

Interestingly, similar to observations in viral infections ^51, 52^, IL-10-producing CD8 ^+^ T cells can be detected in the late elicitation phase of CHS ^53^. Although in respiratory syncytial virus lung infection an autocrine regulatory role of these effector cells has been shown ^52^, in the CHS model this could not be confirmed. Nevertheless, one may speculate that the CD8 ^+^ CHS effector T cells start to express IL-10 later in the elicitation phase and contribute to the limitation of the extent and the downregulation of the CHS response.

The previously reported roles of NKT cells and regulatory B cells (Bregs) in CHS were supported by Fjelbye *et al*. ^54^. These authors showed increased CHS responses in CD1d-deficient mice. This was mostly due to a decrease of IL-10 and a predominant reduction in IL-10 ^+^ Bregs in the spleen and peritoneal cavity. These data strongly suggest a regulation of Bregs by CD1d-restricted NKT cells, which have been identified as an important regulatory cell type in CHS ^55^.

Tolerance to contact allergens can be induced experimentally by different means. Low zone tolerance (LZT) is induced by the repeated epicutaneous application of doses of contact allergens 100- to 1,000-fold lower than the dose used for sensitization. LZT involves IL-10-producing Foxp3 ^+^ Tregs which induce tolerogenic CD8 ^+^CD11c ^+^ DCs and is most likely caused by the presentation of contact allergen to T cells in the absence of skin inflammation ^56^. Another way of tolerance induction is oral tolerance. Hacini-Rachinel *et al*. showed that oral tolerance to DNFB given to mice by gavage was dependent on TLR4 expression on hematopoietic cells ^57^. They demonstrated that TLR4 is necessary to induce the mobilization of tolerogenic CD103 ^+^CD11c ^+^ lamina propria DCs to migrate to the local lymph nodes and prime Foxp3 ^+^ Tregs. Complexes of IL-2/anti-IL-2 antibodies have been shown to enhance IL-2 effects and are considered to treat inflammatory diseases on the basis of their expansion of Tregs. El Beidaq *et al*. demonstrated that tolerance to CHS mediated by TNCB can be enforced by injection of an IL-2/anti-IL-2 antibody reagent, IL-2/JES6-1 ^58^. This treatment, when given before or even after sensitization, established a state of tolerance by CTLA4 ^+^Foxp3 ^+^ Treg expansion that was still evident upon re-challenge with contact allergen even 3 weeks after the last injection. IL-10 and transforming growth factor-beta (TGF-β) levels were increased while neutrophil and CD8 ^+^ T-cell infiltration of the skin were reduced. This study is encouraging, since it shows that a longer-lasting re-establishment of tolerance to contact allergens is possible. The issue of establishing directed, antigen-specific tolerance needs to be solved. However, in a double-blinded, placebo-controlled study, Di Gioacchino *et al*. demonstrated the re-establishment of tolerance to nickel by oral hyposensitization ^59^. A total of 113 patch test-positive patients with systemic nickel syndrome due to nickel-containing foods were given nickel orally for 1 year. Re-introduction of nickel-rich food revealed significant improvement of gastrointestinal symptoms. Skin manifestations also improved, but significance was not reached. On the other end of the spectrum, oral or systemic exposure to contact allergens in sensitized individuals may result in systemic contact dermatitis, a skin reaction characterized by flexural and inverse involvement of eczema. This may occur following high-dose nickel exposure in very nickel-sensitized individuals but also following oral exposure to sesquiterpene lactone allergens (for example, chamomile in tea) in plant-allergic individuals. Other examples include systemic contact dermatitis reactions in patients with an allergy to *Myroxylon pereirae* resin (balsam of Peru) who eat citrus fruits.

## Diagnostics in clinical allergic contact dermatitis

From the clinical point of view, ACD has hardly any specific appearance, although vesicular morphology is frequent and ACD as opposed to ICD characteristically spreads and generalizes if the allergen is not removed. In general, ACD results in an eczematous reaction of the skin, although non-eczematous reactions such as lichenoid reactions and implant failure have also been reported in contact-allergic individuals. This lack of highly specific clinical characteristics indicates the need and benefit of reproducible diagnostic procedures. Since the beginning of clinical contact allergy diagnosis with Jadassohn in 1895, epicutaneous application of suspected contact allergens has been the diagnostic method of choice. The general principle is unchanged since then: contact allergens are dissolved in adequate vehicles such as water, alcohol, and petrolatum, allowing the allergens to penetrate out of the preparation into the epidermis. For most allergens, the highest non-irritating allergen concentrations are optimal ^60^. Classic exceptions are glucocorticosteroids: too high a concentration causes immunosuppression rather than eliciting allergic reactions, although characteristic dermatitis is formed in a ring surrounding the chamber which contains the glucocorticosteroid. For glucocorticosteroids, optimal test concentrations are established on the basis of a more sophisticated balance of sufficient allergen to elicit the allergic reaction and too little to suppress it ^61^. All test preparations for contact allergens are optimized for epicutaneous application, mostly under occlusion, for 48 hours. Patient products may also be applied directly (for example, a piece of a glove or shoe, a metallic disc, nail lacquer, or scrapping from spectacle frames in a chamber). Allergen preparations are kept in place by using adhesive materials with small allergen chambers. The chambers are made from either aluminum or polypropylene and in some products with an inlay from filter paper. The total amount of allergen preparation for one chamber depends on the chamber size. For most of the commercial patch test chambers, 15–20 μL of allergen preparation suffices. Application of too high a volume can result in spoiling out of the test chamber and false-positive patch test reactions. Some allergens are volatile and might evaporate from the patch test material (for example, fragrance chemicals) ^62^. This can occur during the preparation of the patch tests for one patient. Hence, test materials should be prepared directly before application onto the skin. The evaporation process starts for highly volatile allergens directly with manufacturing the test materials. For these allergens, transport and storage in fridges or freezers (especially for isocyanate allergens) are advisable.

In most patients, patch tests are applied onto the back of the patient. After 48 hours of application, patches are removed and test sites are read in accordance with the current guideline ^63^. The readings have to be performed at at least two different time points. Common reading schedules are 48 hours, 72 hours, and 6–7 days. But reading schedules skipping the first reading and including a 96-hour reading are practiced ^64^. The reading of test readings is independent from interpretation of the results, which should be performed subsequently ^65^. Exposure analysis is important before clinical relevance of positive test reactions is decided. Positive test reactions are grouped into current, past, and unknown clinical relevance on the basis of allergen exposure, patient history, and the clinical pattern.

## Current and future treatment options

In the case of clinically relevant ACD, the disease resolves if the patient avoids future skin exposure to the culprit allergen. Thorough information about sources of allergen exposure is crucial, as is instruction about how to read ingredient labels and use spot tests for metal ion release. However, time from avoidance of allergen exposure to complete resolution can take months in the case of severe disease but in milder cases may occur within a few weeks. Topical application of emollients along with anti-inflammatory agents (for example, calcineurin inhibitors and corticosteroids) is first-line treatment and typically will rapidly heal the lesions. However, ACD often occurs in the context of other eczematous conditions, including ICD or atopic dermatitis. In these cases, patients may have a more chronic disease and resolution cannot be obtained through allergen avoidance alone. Here, ultraviolet irradiation or systemic immunosuppressants such as methotrexate, cyclosporine, and azathioprine may be necessary to control inflammation. This is often the case in patients with chronic hand eczema. Systemic corticosteroids should generally be avoided in patients with chronic dermatitis because of the risk of severe complication such as osteoporosis and type 2 diabetes but may be very useful in patients with acute severe ACD following exposure to, for example, poison ivy or para-phenylenediamine (PPD). So far, no biologics have been proven to be useful in the treatment of ACD, although only case series have been performed ^66^. Based on the successful experience of patch testing patients who receive various biologics and immunosuppressants, none of these seems to really suppress ACD and therefore they are not suitable for treating ACD per se ^67^. So far, oral tolerance induction has not been successful, but a randomized Italian study showed that an improvement of gastrointestinal symptoms following oral nickel exposure by food and a significant increase in patch test negativity may be obtained ^59^. At the moment, it is impossible to envision any drugs that may directly inhibit ACD responses in affected patients, but it is possible that the innate immune system needs to be targeted at an early stage to prevent the cascade of reactions in chronic disease ^9^.

Given the complexity of ACD, a multi-layered strategy for treatment seems necessary. Interference with mechanisms of inflammation, enforcement of immune regulation, and reduction, removal, or suppression of effector and memory T cells, including T _RM_ cells, are areas to be exploited in the future. Based on our increasing understanding of the pathomechanisms, combination therapies may have to be developed for acute and hard-to-treat chronic ACD. The ongoing search for biomarkers will hopefully lead to the identification of profiles suitable for modern molecular diagnostics. New *in vitro* assays should be suitable to identify contact allergens for hazard identification ^10^. Here, assays that allow potency assessment of contact allergens are urgently needed.

The ongoing basic and clinical research as well as *in vitro* testing for contact allergen identification, the recognition of the impact of contact dermatitis, and the continuing education and training to raise awareness for prevention and for improvement of workplaces ^5^ will result in avoidance of hazardous chemicals and improved management of this important skin disease.

## Abbreviations

ACD, allergic contact dermatitis; BMDC, bone marrow-derived dendritic cell; Breg, regulatory B cell; CHS, contact hypersensitivity; DC, dendritic cell; DETC, dendritic epidermal T cell; DNCB, 2,4-dinitrochlorobenzene; DNFB, 2,4-dinitrofluorobenzene; ICD, irritant contact dermatitis; IFN-γ, interferon-gamma; IL, interleukin; KO, knockout; LZT, low zone tolerance; MIF, macrophage migration inhibition factor; NK, natural killer; NKT, natural killer T; NLRP3, NOD-like receptor pyrin containing 3; OECD, Organisation for Economic Co-operation and Development; TLR, Toll-like receptor; TNCB, 2,4,6-trinitrofluorobenzene; Treg, regulatory T cell; T _RM_, tissue-resident memory T
